# Using a positive deviance framework to identify Local Health Departments in Communities with exceptional maternal and child health outcomes: a cross sectional study

**DOI:** 10.1186/s12889-016-3259-7

**Published:** 2016-07-19

**Authors:** Tamar Klaiman, Athena Pantazis, Anjali Chainani, Betty Bekemeier

**Affiliations:** AccessMatters, 1700 Market St., Suite 15th Fl., Philadelphia, PA 19103 USA; University of Washington School of Nursing, Psychosocial & Community Health, Box 357263, Seattle, WA USA; University of the Sciences, 4101 Woodland Ave., Box 22, Philadelphia, PA 19104 USA

**Keywords:** Positive deviance, Local health departments, Outcomes, Methods

## Abstract

**Background:**

The United States spends more than most other countries per capita on maternal and child health (MCH), and yet lags behind other countries in MCH outcomes. Local health departments (LHDs) are responsible for administering various maternal and child health programs and interventions, especially to vulnerable populations. The goal of this study was to identify local health department jurisdictions (LHDs) that had exceptional maternal and child health outcomes compared to their in-state peers – positive deviants (PDs) - in Washington, Florida and New York in order to support the identification of strategies that can improve community health outcomes.

**Methods:**

We used MCH expenditure data for all LHDs in FL (*n* = 67), and WA (*n* = 35), and most LHDs in NY (*n* = 48) for 2009–2010 from the Public Health Activities and Services Tracking (PHAST) database. We conducted our analysis in 2014–2015. Data were linked with variables depicting local context and LHD structure. We used a cross-sectional study design to identify communities with better than expected MCH outcomes and multiple regression analysis to control for factors outside of and within LHD control.

**Results:**

We identified 50 positive deviant LHD jurisdictions across 3 states: WA = 10 (29 %); FL = 24 (36 %); NY = 16 (33 %). Overall, internal factor variables improved model fit for identifying PD LHD jurisdictions, but individual variables were not significant.

**Conclusions:**

We empirically identified LHD jurisdictions with better MCH outcomes compared to their peers. Research is needed to assess what factors contributed to these exceptional MCH outcomes and over which LHDs have control. The positive deviance method we used to identify high performing local health jurisdictions in the area of maternal and child health outcomes can assist in better understanding what practices work to improve health outcomes. We found that funding may not be the only predictor of exceptional outcomes, but rather, there may be activities that positive deviant LHDs are conducting that lead to improved outcomes, even during difficult financial circumstances. This method can be applied to other outcomes, communities, and/or services.

## Background

The U.S. spends more on maternal and child health (MCH) services than most of the world; [1] however, MCH outcomes in the U.S. lag behind most other industrialized nations [2]. Unintended pregnancies, low-birth weight and pre-term births, high risk health behaviors, socio-economic factors and access to health services all contribute to the short and long-term health of families in our communities [3]. Local health departments (LHDs) in the U.S. often administer MCH services including Special Supplemental Nutrition Program for Women, Infants, and Children (WIC), home visiting, and prenatal care provider referral; as well as education regarding healthy pregnancies, breastfeeding, and postpartum care. While many of these services have been shown to reduce disparities and improve outcomes, [4, 5] the formula for establishing and maintaining good community-level MCH outcomes remains elusive, resulting in tremendous variation in local public health system service delivery and a lack of evidence to support best practices and guide local public health leaders [6, 7].

Mays, et al. noted that the ability of LHDs to meet community needs varies with spending levels, identifying funding as a major contributor to LHD performance [8]. This evidence, however, has been insufficient to lead to adequate or consistent funding across LHDs as public health funding levels and practices remain extremely variable [6]. Fiscal constraints in the current economic climate have also led to drastic public health workforce job losses and the elimination and reduction of MCH services in many communities [9].

LHD leaders, nonetheless, use a variety of strategies to minimize negative financial impacts, [10] with some of these LHDs achieving a particularly high level of performance compared to peer communities [11]. A recent study found that strong public health system partnerships could increase the provision of public health services to the community [12]. There is also some evidence that certain leadership characteristics, financial resources, and a well-educated staff lead to better community health outcomes [13, 14]. While LHD leaders may know that increased funding, staff, training, and community support can lead to better outcomes; inadequate evidence exists regarding how they might leverage these (or other) factors to achieve better health outcomes with less than optimal resources. Empirically identifying LHD jurisdictions with exceptionally positive health outcomes and examining their practices could help all public health system leaders optimize performance and improve population health during turbulent financial times. The aim of this project was to identify local health department jurisdictions (LHDs) that had exceptional MCH outcomes compared to their in-state peers – positive deviants (PDs) - in Washington, Florida and New York. Additionally, we sought to identify factors that were associated with exceptional MCH outcomes.

### Positive deviance

Since the articulation of the ten essential public health services, researchers have used a variety of strategies to assess public health system performance including grant reporting requirements, program evaluations, and community health assessments [15]. These assessment tools, however, tend to rely on self-reported, often incomplete data that may not be externally validated. In our study, we used a systematic means of rigorously identifying LHDs in communities with better than expected MCH outcomes compared to peers in their state – positive deviants – in order to determine what factors support positive unexpected population-level achievements.

Positive deviance is a framework that identifies and learns from units that perform beyond expectations. In public health, positive deviance originated from the concept that interventions could be designed around uncommon beneficial health behaviors that some community members already practiced, [16] and more recently, to identify system-level approaches to improve population health outcomes by reducing unwarranted variability between or within institutions [17].

### Realist evaluation

According to Pawson [18] a primary function of evaluation research should be to capture a clear understanding of why some programs achieve better outcomes than others. Traditional evaluation approaches such as meta-analysis and narrative review are often either so general as to make no effort to understand how programs work contextually, or are so specific to times and places that lessons cannot be transferred to other contexts [18]. To overcome these limitations, evaluation research should seek to understand program mechanisms, put findings in context, and generalize findings to external situations (Context + Mechanism = Outcome). We utilized this framework to organize the methods for identifying positive deviant LHD jurisdictions in order to explain not only who was performing well, but *why.*

Numerous factors influence population health outcomes, over which LHD administrators have varying levels of immediate control. Factors over which LHD leaders have limited or no control - the *context* in which they find themselves [19] - may include state and local budgets, population size, and geography. Many of these factors have been found to have a significant impact on LHD performance [8, 20]. LHD staff, however, cannot generally change these circumstances.

Factors over which LHD leaders have some control include the activities they choose to undertake - *mechanisms* [19] used to improve health outcomes - including establishing partnerships, applying for external funding, and choosing which services and how much of them to provide in the community. Many of these variables have been shown to impact community health outcomes and are much easier to change than context factors.

*Outcomes* result from a combination of *context* and *mechanisms (context + mechanism = outcome)* [19]. We identified LHDs in communities that experienced better outcomes than their in-state peers to better understand the interaction between the environments in which LHDs exist (context) and how specific activities are triggered (mechanisms) that lead to exceptional health outcomes. The positive deviance approach can help improve the performance of public health systems by building a shared evidence-base of best practices to improve outcomes across institutions and geographic locations, while taking into consideration local context and resources.

## Methods

We defined positive deviants as LHD jurisdictions with better MCH outcomes compared to peers within their state. We used data compiled within the Public Health Activities and Services Tracking (PHAST) database. The Robert Wood Johnson Foundation (RWJF)-funded PHAST study team (PI. B. Bekemeier) has compiled externally validated measures of public health service production in key public health priority areas including MCH [21].

PHAST is a multidisciplinary, practice-based research collaborative developed in relation to RWJF’s national system of Public Health Practice-Based Research Networks (PBRNs) [22]. The PHAST research team, together with PBRN practice partners, has developed a uniquely detailed comprehensive, accessible database from administrative data and depicting variations in LHD practice and funding [23].

We conducted a cross sectional study, and we included complete and fully linked annual data from 2009 to 2010 for all LHD jurisdictions in FL (*n* = 67) and WA (*n* = 35) and most jurisdictions in NY (*n* = 48) in our study. We conducted our analysis in 2014–2015 using the most recent data available. Additionally the PHAST database only had complete data available for 2009–2010 and for the three states we included in the analysis. A PHAST dataset similar to ours was used in a study of associations between LHD MCH expenditures and health outcomes [23]. We excluded New York City because the size and budget of the New York City Health Department are dramatically disproportionate to other NY LHDs. We also excluded nine additional NY jurisdictions that had missing data. All 150 LHDs in our sample (300 over 2 years) served single county jurisdiction. Drawing data from the PHAST dataset assured that these detailed LHD data had been thoroughly processed and documented, along with review and clarification by practice partners in the relevant states [24]. The University of the Sciences Institutional Review Board ruled this project exempt.

### Variables

We used multivariate regression models to identify positive deviant LHDs jurisdictions, in relation to MCH outcomes. Our goal was to exploit the linear relationship between contributing factors and outcomes, [21] rather than test hypotheses or make inferences. The model we used sought to explain MCH outcomes (Y) as a function of contextual factors (Z) (over which LHDs have little or no control) and internal mechanisms (X) (over which LHDs have some control [18].

We included the following contextual variables in our analysis (Appendix): total LHD expenditures, county level population size, number of Medicaid-funded births, Core-Based Statistical Area (CBSA) (metropolitan, micropolitan, rural), [25] Robert and Reither’s Social Disadvantage Index, [26] percent of children under 18 living in poverty, percent of persons 25+ with high school or more education, percent of African American residents, percent of Hispanic residents, and per capita health care providers (nurses, midwives, and doctors). Similar variables have been used as controls in other PHAST MCH studies [21, 24]. As controllable mechanisms (X), that have been shown to be related to LHD service provision and/or health outcomes, [27] we included whether or not certain public health services are being provided instead of - or also by - an “alternative provider” [21, 28] and if the LHD’s lead executive is a clinician [13, 29]. We also classified LHDs based on a latent measure developed from activities described as provided by individual LHDs in the 2008 National Association of County and City Health Officials (NACCHO) National Profile of Local Health Departments (*Profile*) survey [30]. The latent measure developed from the *Profile* capture an LHD’s general approach to service delivery as in a range of overall service delivery: limited to comprehensive [31]. For outcomes (Y) we included rates of teenage pregnancy/births, rates of late or no prenatal care, infant mortality rate, and percent of low weight births. These are proximal MCH outcomes where relationships between investments and outcomes have been found [21].

### Multivariate analysis

Identifying positive deviants required classifying all cases exceeding or not exceeding a threshold for each outcome [32]. We defined positive deviants using three steps:

- **Step 1**: We regressed *Y = α +* ***β***_***1***_*(Z) + e* to identify potential positive deviants in each outcome taking into account local contextual factors.
- **Step 2**: We added in X variables *Y = α +* ***β***_***1***_*(Z) +* ***β***_***2***_*(X) + e* to assess how well the models fit when including LHD-controlled variables.
- **Step 3**: We conducted a likelihood ratio test (LRT) to evaluate whether the inclusion of the additional variables based on theoretical considerations improved model fit. If the coefficients for the X variables ***β***_***2***_ ≠ 0, we examined residuals (e) from the regression in step 2, therefore taking advantage of the variance explained by measurable factors in refining our identification of outliers. ***β***_***2***_ ≠ 0 for all of our models except two outcomes in NY where the LRT indicated that ***β***_***2***_ did not add anything (Table 1). However, for the sake of consistency with the other analyses, we included the full models for these New York outcomes as well. The data analyses were run for each outcome and run for both years [21].

**Table 1:** Positive deviant identification regression results

State	Model outcomes	R^2^		Likeilhood ratio test
		Step 1	Step 2	*p-value*
Florida	Teen pregnancy rate	0.65	0.69	0.001
	Infant Mortality rate	0.23	0.27	0.03
	Late or no prenatal care rate	0.10	0.20	0.002
	Low birth weight rate	0.45	0.52	< 0.001
New York	Teen pregnancy rate	0.50	0.51	0.17
	Infant Mortality rate	0.32	0.33	0.12
	Late or no prenatal care rate	0.55	0.65	< 0.001
	Low birth weight rate	0.28	0.39	0.001
Washington	Teen pregnancy rate	0.82	0.84	0.005
	Infant Mortality rate	0.22	0.33	0.005
	Late or no prenatal care rate	0.33	0.53	< 0.001
	Low birth weight rate	0.30	0.50	< 0.001

Examining each outcome where lower was better; we identified potential positive deviants as those with studentized residuals less than −1, as they performed better in the outcome than the model predicted (better than their peers). Using a cutoff of −2 was too restrictive and did not allow for variation in LHD context. We identified influential leverage points (*n* = 4, 1 %) to ensure we were obtaining the same positive deviants with and without them. The analysis was robust to the removal of leverage points except for two LHDs each in 2009 and 2010 which were removed from the Florida models for low birth weight, infant mortality rate and late or no prenatal care. The positive deviant observations we identified were largely robust to sensitivity analyses with and without these influential leverage points. The few that were not robust^1^ had relationships between the outcome and inputs that were consistent with the overall trend. We ran additional sensitivity analyses to ensure that LHDs with missing data were similar to those included in the models and that selection of positive deviants was robust to removal of highly correlated covariates and to the use of more parsimonious models.

We conducted these analyses within states for each outcome. This allowed us to control for state-level differences in expenditure and outcome measures and created a consistent scale for identifying state-specific outliers. To ensure reliability in our identified positive deviant observations, we created a matrix and included only LHDs that were identified as outliers over multiple years and/or outcomes. An LHD, for example, that had exceptional outcomes in low birth weight in 2009, and low birth weight and low teenage birth rate in 2010 would be included, but an LHD with only exceptional rates of low birth rate in 2009 would not be included.

## Results

We identified a total of 50 LHD jurisdictions out of 150 LHDs per year (300 over two years), across 3 states: WA = 10 (29 %); FL = 24 (36 %); NY = 16 (33 %). Forty-six of 50 LHDs (92 %) performed better than expected in at least one MCH outcome over 2 years, and 32 LHDs (64 %) had 2 or more exceptional outcomes in a single study year. Positive deviant LHD jurisdictions varied by community-type similarly to the pool of LHDs we included in our study: 28 % of positive deviant LHD jurisdictions were from rural communities, 20 % micropolitan, and 52 % metropolitan compared to 25, 23, and 52 % of all LHD jurisdictions respectively. An advisory council reviewed our findings to ensure face validity of the results.

Initial analysis showed that none of the *context* variables were significantly associated with the outcomes of interest (Table 1). Specifically, we expected LHD funding to be associated with better MCH outcomes; however, we found great variations in expenditures between LHDs. In ten circumstances average per capita spending by positive deviant jurisdictions was less than non-positive deviant counterparts (Table 2). FL rural positive deviant LHDs, for example, spent less on average on WIC and on family planning than other FL LHDs. In NY, rural positive deviant LHDs spent less on average than non-positive deviant LHDs on per capita Maternal/Infant/Child/Adolescent Health (MICA) expenditures [24]. In WA, micropolitan positive deviant LHDs averaged less on total MCH expenditures, WIC expenditures, and family planning expenditures than their metropolitan and rural counterparts. Metropolitan positive deviant LHDs in WA also spent less on average on total MCH expenditures, family planning expenditures, and MICA expenditures than their non-positive deviant metropolitan counterparts. Across all three states in our sample, average LHD expenditures on WIC were consistently lower in positive deviant LHDs in rural communities.

**Table 2:** MCH Expenditures by LHD context

		Type of LHD		Per capita total maternal child health expenditures		Per capita WIC expenditures		Per capita family planning expenditures		Per capita maternal, infant, child and adolescent health expenditures	
State		*non-PDs*	*PDs*	*non-PDs*	*PDs*	*non-PDs*	*PDs*	*non-PDs*	*PDs*	*non-PDs*	*PDs*
FL	*Rural*	18 (27 %)	7 (29 %)	$ 5.78–35.67 (19.68)	$ 7.64–33.26 (22.71)	**$ 0–21.20 (1.91)**	**$ 0–0.89 (0.22)**	**$ 4.49**–**15.42 (9.35)**	**$ 2.38**–**16.03 (8.49)**	$ 0.01–23.60 (8.42)	$ 4.48–22.41 (14.00)
	*Micro*	10 (15 %)	2 (8 %)	$ 8.56–46.36 (20.80)	$ 28.05–36.26 (32.98)	$ 0.02–11.45 (4.80)	$ 0.02–11.05 (5.52)	$ 4.01–15.84 (6.27)	$ 9.12–20.72 (14.13)	$ 0.06–30.82 (9.73)	$ 10.57–16.09 (13.33)
	*Metro*	39 (58 %)	15 (63 %)	$ 7.26–27.69 (15.49)	$ 7.49–56.38 (16.93)	$ 0–11.89 (5.40)	$ 0.02–15.01 (5.15)	$ 1.22–9.59 (4.06)	$ 1.97–10.87 (4.33)	$ 0.26–16.85 (6.02)	$ 0.32–32.04 (7.44)
NY	*Rural*	9 (19 %)	4 (25 %)	$ 0.25–14.06 (5.77)	$ 1.18–16.61 (7.94)	$ 0–8.70 (1.76)	$ 0.26–7.48 (2.42)	$ 0–13.87 (2.54)	$ 0.03–8.77 (4.46)	**$0.10**–**6.13 (1.47)**	**$ 0.04**–**3.03 (1.06)**
	*Micro*	13 (27 %)	5 (31 %)	$ 0.30–12.90 (2.56)	$ 1.38–20.55 (9.92)	$ 0.01–8.05 (1.40)	$ 0.12–10.12 (3.28)	$ 0–6.52 (0.43)	$0.04–17.37 (4.75)	$ 0.08–2.41 (0.72)	$ 0.24–3.62 (1.89)
	*Metro*	26 (54 %)	7 (44 %)	$ 0.02–13.70 (4.81)	$ 1.07–20.39 (7.50)	$ 0–7.77 (2.28)	$ 0–6.54 (3.71)	$ 0–3.11 (0.30)	$ 0–3.18 (0.62)	$ 0–8.31 (2.22)	$ 0.86–11.14 (3.17)
WA	*Rural*	11 (31 %)	3 (30 %)	$ 3.44–32.20 (15.16)	$ 17.17–25.95 (21.22)	$ 0–8.68 (3.96)	$ 4.98–8.97 (7.31)	$ 0–17.86 (3.84)	$ 0–10.27 (5.55)	$ 2.36–18.83 (7.37)	$ 3.14–11.81 (8.36)
	*Micro*	11 (31 %)	3 (30 %)	**$ 1.21**–**9.40 (5.77)**	**$ 2.36**–**6.21 (4.48)**	**$ 0–5.33 (2.90)**	**$ 0–3.43 (1.55)**	**$ 0–0.64 (0.08)**	**$ 0–0.01 (0)**	$ 1.02–4.67 (2.79)	$ 1.09–5.11 (2.92)
	*Metro*	13 (37 %)	4 (40 %)	**$ 0.82**–**27.52 (9.30)**	**$ 0.73**–**11.71 (7.32)**	$ 0–4.71 (1.78)	$ 0–4.98 (2.76)	**$ 0–10.09 (2.15)**	**$ 0–2.87 (1.14)**	**$ 0.82**–**18.78 (5.36)**	**$ 0.73**–**5.36 (3.42)**
Combined	*Rural*	38 (25 %)	14 (28 %)	$0.25–35.67 (15.44)	$1.18–33.21 (17.68)	**$ 0–21.20 (2.56)**	**$ 0–8.97 (2.34)**	$ 0–17.86 (6.18)	$ 0–16.03 (6.61)	$ 0.01–23.60 (6.71)	$ 0.04–22.41 (8.73)
	*Micro*	34 (23 %)	10 (20 %)	$0.30–46.36 (9.72)	$ 1.38–35.26 (13.05)	$ 0–11.45 (3.00)	$ 0–11.05 (3.21)	$ 0–15.84 (2.31)	$ 0–20.72 (5.23)	$ 0.06–30.82 (4.40)	$ 0.23–16.09 (4.62)
	*Metro*	78 (52 %)	26 (52 %)	$ 0.17–27.69 (10.50)	$0.73–56.37 (13.00)	$ 0–11.87 (3.64)	$ 0–15.01 (4.40)	$ 0–10.09 (2.36)	$ 0–10.87 (2.86)	$ 0.01–18.78 (4.50)	$ 0.32–32.04 (5.75)

Bold = PDs spent less than non-PDs on average

The addition of the *mechanism* variables - whether or not certain public health services are being provided instead of - or also by - an “alternative provider”; if the LHD’s lead executive is a clinician, and a latent service delivery measure - to the *context* variable models added significant improvements to model fit in almost all cases, but none of the individual variables were significant (Table 1). This offered indications that *mechanisms* had some influence on outcomes, but our existing data and sample size did not support the generation of clear explanations regarding the action of these *mechanisms* or of other influences on MCH outcomes.

## Discussion

Our study aim was to identify local health department jurisdictions (LHDs) that had exceptional maternal and child health outcomes compared to their in-state peers – positive deviants (PDs) - in Washington, Florida and New York. Additionally, we hoped to identify the combination of *context* and *mechanisms* that led to exceptional MCH *outcomes. Context* variables were not as clearly associated with exceptional outcomes as we would have expected. This study underscores the high level of variation that exists across public health systems in the U.S., particularly with regard to MCH expenditures across geographic locations [21]. The financial *context* of LHD expenditures, while important to jurisdictions’ health outcomes, [8, 21] is seemingly not the sole predictor. There may, in fact, be other factors that may not be dependent upon financial investments that are associated with improved outcomes, This is particularly important as financial resources for public health are increasingly constrained.

We also found that the *mechanisms* used by exceptional LHD jurisdictions improved model fit for identifying positive deviants; however, none of the individual *mechanism* variables were significant. This suggests that in-depth study of the specific *mechanisms* used by LHDs in these exceptional communities could point to underlying best practices that we did not measure and that may not currently be well understood among researchers and practice leaders.

LHDs in positive deviant communities may have strong partnerships with local providers and community-based organizations that have led to exceptional outcomes. There is some evidence that partnerships may be associated with better health outcomes and LHD performance; [33] however, robust metrics for studying the strength of LHD partnerships have yet to be developed. Prybil et al. recently noted best practices in hospital-public health collaborations, [34] some of which may be occurring in these positive deviant communities. While some positive deviant jurisdictions in our study may not have a hospital, they may be applying partnership strategies with private providers and community based organizations, leading to exceptional outcomes.

A recent issue of the *American Journal of Preventive Medicine* focused entirely on public health workforce issues and highlighted the need for a better-trained workforce capable of effectively addressing community health needs in a highly volatile public health environment [35]. Many LHDs are changing the types of services they provide [9] requiring LHD staff to learn new skills to adjust to changing priorities [10]. Positive deviant jurisdictions in our study, particularly those that experienced exceptional MCH outcomes while spending less money, may be those with LHDs that are addressing workforce issues by training staff and developing leadership toward a more population-focused practice that assures effective service delivery and community conditions that promote health. These may be LHDs that are spending less on WIC service delivery, for example, and working instead to facilitate WIC service delivery among other local providers.

Some LHDs in positive deviant jurisdictions may also be using robust systems of data collection and reporting to identify MCH priorities specific to their communities. Nationally, most LHDs conduct community health assessments, [36] but the metrics used to assess population health vary greatly and suggest some need for standardization. Widely comparing how LHDs and communities use of data for assessment and decision-making across jurisdictions is currently a challenge, but an in-depth examination of how data are accessed and used by high performing LHDs would help inform assessment processes and could lead to improved collection and use of data.

Additional research about the combination of *context* and *mechanisms* that has led to exceptional *outcomes* in the jurisdictions we identified is essential for identifying these modifiable factors and best practices in MCH*.* An in-depth analyses of these positive deviant jurisdictions would generate evidence regarding *why* some communities have better outcomes, even in resource-constrained environments, and provide critical guidance to LHD leaders and community partners in other jurisdictions. This finding led us to conduct in-depth interviews with LHD staff to learn more about activities that may influence MCH outcomes but were not captured in the quantitative analysis [37].

Limitations of our study include regression models that may have been underpowered given our small sample size. Our approach helped mitigate this by including all LHDs with complete data for each study state. Our quantitative analysis may also have led to inclusion of some LHDs that met our cut off of exceptional by chance. We erred on the side of inclusion in order to have sufficient examples of positive deviants by community type as small, rural LHDs are often excluded from analyses of LHD activities. The cross sectional nature of our study limited the ability to consider high performers over time; however comparing within states allowed for some parity in the comparisons. To obtain as robust a sample as possible, we included only LHDs that were positive deviants for multiple outcomes and/or years. We intend to update the analysis with additional years of data and additional states in the future.

## Conclusion

In this project, we identified local health department jurisdictions (LHDs) that had exceptional maternal and child health outcomes compared to their in-state peers – positive deviants (PDs) - in order to support the identification of strategies that can improve community health outcomes. Our research demonstrated a means to empirically identify communities with exceptional MCH outcomes while taking local context into consideration. Such identification lays the groundwork for conducting in-depth analyses with positive deviant LHDs in order to understand what local MCH practices lead to exceptional outcomes. The empirical positive deviance method presented here offers an initial and necessary step in discovering the kinds of practices high performing LHDs and their communities have in place that lead to better MCH outcomes than their peers. Further study is needed to identify and measure the *mechanisms* that lead to exceptional outcomes.

## Abbreviations

LHD, Local Health Department; MCH, maternal and child health; WIC, Special Supplemental Nutrition Program for Women, Infants, and Children (WIC)

## Appendix

**Table 3:** Variables included in the regression models

Variables	Notes
*External factors LHDs cannot control (Context)*	
Total LHD expenditure	Raw number or per capita
Population	Total population of the county/area the LHD serves (number)
Number of Medicaid births in the county	Controls for need of services
Core Based Statistical Area (CSBA) [28]	3 levels: metropolitan, micropolitan, rural
% of children under 18 living in poverty	
Social Disadvantage Index [29]	Index of median HH income; % of households receiving public assistance; % unemployed
% of persons 25+ with HS or more education in county	
% of county population that is African American	
% of county population that is Hispanic	
Per Capita number of nurses	
Per Capita number of midwives	
Per Capita number of doctors	
*Inputs (LHD can control)*	
Alternative Provider	Categorical variables for each MCH service area (linked to expenditure included); captures whether other entity is providing the service in the county area
Executive Clinician	0-1; captures whether the executive at the LHD has a clinical degree or not
LHD Service Approach Classification	Categorical variable (4 levels) obtained through latent class analysis of self-reported LHD service provisions to the NACCHO profile survey [34]
*MCH Outcomes*	
Teen pregnancy rate	Number of births to girls age 15–19 over total number of girls age 15–19 (× 1000), smoothed (3 year average)
Infant Mortality rate	Number of infant deaths over total number of births, smoothed (3 year average)
Late or no prenatal care rate	Number of infants born that received no or late prenatal care over total births, smoothed (3 year average)
Low birth weight rate	Number of infants born at low birth weight over total births, smoothed (3 year average)

## Footnotes

1 These all occurred in Florida (total *n*=67 per year): *n*=2 for low birth weight in 2009, 2 in 2010; *n*=1 for infant mortality rate in 2010; *n*=4 for prenatal care in 2009, 1 in 2010; and *n*=2 for teen birth rate in 2009, 3 in 2010.
